# *Amy2B* copy number variation reveals starch diet adaptations in ancient European dogs

**DOI:** 10.1098/rsos.160449

**Published:** 2016-11-09

**Authors:** Morgane Ollivier, Anne Tresset, Fabiola Bastian, Laetitia Lagoutte, Erik Axelsson, Maja-Louise Arendt, Adrian Bălăşescu, Marjan Marshour, Mikhail V. Sablin, Laure Salanova, Jean-Denis Vigne, Christophe Hitte, Catherine Hänni

**Affiliations:** 1 CNRS/ENS de Lyon, French National Platform of Paleogenetics, PALGENE, Ecole Normale Supérieure de Lyon, 46 allée d'Italie, 69364 Lyon Cedex 07, France; 2 Laboratoire d'Ecologie Alpine (LECA), Université Grenoble Alpes, 38000 Grenoble, France; 3 CNRS/MNHN/SUs-UMR 7209 Archéozoologie, Archéobotanique: Sociétés, Pratiques et Environnements, 55 rue Buffon, 75005 Paris, France; 4 Institut de Génétique et Développement de Rennes, CNRS-UMR6290, Université de Rennes1, 35000 Rennes, France; 5 Science for Life Laboratory, Department of Medical Biochemistry and Microbiology, Uppsala University, 75237 Uppsala, Sweden; 6 The National Museum of Romanian History, 12 Calea Victoriei, 030026 Bucharest, Romania; 7 Russian Academy of Science, Zoological Institute, Saint Petersburg, Russia; 8 CNRS/ENS, Aoroc, 45 rue d'Ulm, 75005 Paris, France

**Keywords:** domestication, palaeogenomics, amylase, dog, Neolithic

## Abstract

Extant dog and wolf DNA indicates that dog domestication was accompanied by the selection of a series of duplications on the *Amy2B* gene coding for pancreatic amylase. In this study, we used a palaeogenetic approach to investigate the timing and expansion of the *Amy2B* gene in the ancient dog populations of Western and Eastern Europe and Southwest Asia. Quantitative polymerase chain reaction was used to estimate the copy numbers of this gene for 13 ancient dog samples, dated to between 15 000 and 4000 years before present (cal. BP). This evidenced an increase of *Amy2B* copies in ancient dogs from as early as the 7th millennium cal. BP in Southeastern Europe. We found that the gene expansion was not fixed across all dogs within this early farming context, with ancient dogs bearing between 2 and 20 diploid copies of the gene. The results also suggested that selection for the increased *Amy2B* copy number started 7000 years cal. BP, at the latest. This expansion reflects a local adaptation that allowed dogs to thrive on a starch rich diet, especially within early farming societies, and suggests a biocultural coevolution of dog genes and human culture.

## 1. Introduction

In western Eurasia, the Neolithic transition took place between 11 500 and 6000 cal. BP (before present), leading to the shift from hunting and gathering to farming [1,2]. At this time, the dog, domesticated during the Upper Palaeolithic [3–6], had accompanied humans for several millennia. The antiquity of this close proximity has already been highlighted by archaeological and genomic approaches [4,7–11]; but the impact of human lifestyle and diet changes on dog genetic characteristics, during the Neolithic transition, is still being investigated. This is of crucial importance in understanding the development of early farming societies, early domestic canid physiological changes and the genomic transformations towards modern dog genotypes and phenotypes.

According to the morphology of archaeological specimens, the genomics of modern canids and experimental domestications, early dogs experienced selective pressures involving behavioural, morphological and physiological traits [3,10–15]. A comparison of genome-wide patterns of genetic variation from a large group of dogs and wolves identified genomic regions affected by directional selection during dog domestication [16]. These included several genes involved in digestion and energy metabolism, most likely connected to a diet change in the dog's lineages [16]. In particular, it was noted that selection had targeted a series of duplications of the gene coding for pancreatic amylase (*Amy2B*). This led to a several fold copy number increase in modern dog breeds in comparison with their wild ancestor, the wolf, that is associated with a higher amylase activity [16,17]. Whereas *Amy2B* copy numbers vary widely in dogs (4–34 copies), both at a breed and individual level [18], the copy number range is much lower (two to eight copies) across wolf populations with 60% of the wolves bearing only two copies [17]. This suggests that dogs have adapted to a diet richer in starch, relative to the carnivorous wolf diet [16].

Present-day canids present three patterns with regard to the *Amy2B* copy number variation [16,17]: (i) 60% of the wolves, and most of the dingos bear two copies of the gene, (ii) a second pattern shows dogs and wolves with two to eight copies of *Amy2B*, and (iii) and a third pattern consists of dogs that bear more than eight copies of *Amy2B*.

The question of a link between the increase of the *Amy2B* copy number in dogs and the Neolithic transition has been previously raised [16–18]. However, this question remains unanswered as we can only hypothesize that the increase could have provided a strong adaptive advantage within a farming context, and we cannot exclude that it occurred much later, as a result of the more recent selection of specialized lineages [19–21].

Palaeogenetics provides a unique opportunity to shed light on this question by investigating the landscape relative to the *Amy2B* copy number variation in ancient canid populations. In this study, we examined the *Amy2B* copy number of ancient Eurasian dogs by highlighting the *Amy2B* gene expansion from the 7th millennium cal. BP to the Bronze Age (*ca* 4000 cal. BP). We showed *Amy2B* gene expansion in dog samples coming from archaeological sites corresponding to early farming contexts located in Western Europe, Southeastern Europe and Southwest Asia.

## 2. Material and methods

### 2.1. Dog morphotype samples

We attempted to study the *Amy2B* copy number, using ancient DNA (aDNA) analysis from the tooth and bone remains of 88 different canids from 30 archaeological sites in Western Europe, Romania, Russia, Estonia, Israel, Turkmenistan and the Iranian Plateau, from the Upper Palaeolithic to the Bronze Age. In total, aDNA results were obtained from 13 individuals from eight archaeological sites in Europe and Turkmenistan (see the electronic supplementary material). The osteological distinction between the domestic dog (*Canis familiaris*) and the wolf (*Canis lupus*), its wild ancestor, can be difficult, due to the regional and temporal variability of wolf morphology [22] and to the morphological proximity between the two forms in the early steps of domestication; therefore, we used a series of osteological traits to separate them [3,23,24]. Dogs differ from wolves by their overall significantly smaller size, a smaller brain-case volume, a shorter snout, tooth crowding and a higher frequency of dental defects. All the individuals used in this study belonged to the domestic form, according to one or several of these criteria.

When possible, measurements were taken from mandibles, particularly the five dimensions frequently measurable in broken archaeological specimens (dimensions #8, 10, 11, 19, 20, after [25]; electronic supplementary material, table S1—only measurements for individuals providing aDNA results are reported). The data obtained for our archaeological Holocene canids were then compared with the data derived from (i) a series of Pleistocene wolf mandibles from Arcy-sur-Cure (France) [23] dated between 100 000 and 60 000 years BP, prior to any suspicion of domestication; (ii) a series of Pleistocene canid mandibles from Předmostí (Czech Republic) [26], attributed to the wolf and dated to 27 000–26 000 BP; (iii) a series of modern Eurasian wolf mandibles from the National Museum of Natural History, Paris [23]; and (iv) a series of modern wolf mandibles from Southeastern Europe [27] (electronic supplementary material, figure S1). It was noted that the length of the tooth row (dimension #8 [25]) was significantly different between the Holocene canids and the four series of wolf (Mann–Whitney tests corrected for Bonferroni, *p *< 0.05). The only individual in the Holocene series, located at the very margin of the modern wolves' variation interval (CH1075; electronic supplementary material, figure S1), evidenced a colour mutation typical to domestic animals from one of our previous studies on the same material [15]. Therefore, the canid series analysed in this study can be identified to be the domestic form *C. familiaris*.

### 2.2. Ancient DNA

All the aDNA procedures were conducted at the French National Platform of Paleogenetics (PALGENE, CNRS, ENS de Lyon) using facilities and tools specific to aDNA analyses, while following adequate controls [28–33].

#### 2.2.1. Ancient DNA extraction

The external surface of the bones was scratched with a sterile scalpel to produce a clean piece, which was then reduced to powder with a sterile hammer. The powder (150–300 mg) was then digested for 18 h at 55°C with agitation in 4.7 ml of buffer (0.5 M EDTA (ethylene diamine tetra acetic acid), pH = 8.0), 50 µl of proteinase K (1 mg ml^−1^) and 250 µl of 0.5% *N*-lauryl-sarcosyl [28]. A silica-based method modified from Rohland & Hofreiter [32] was used to retrieve the aDNA. Mock extractions were performed in order to rule out contamination from reagents. In addition, cross-contamination was monitored by combining the aDNA from our samples with the aDNA from other species (i.e. owls, fish and sheep) for each extraction session.

#### 2.2.2. Ancient DNA pre-amplification and quantitation

In order to restore sufficient aDNA quantity for each sample, we co-amplified the nuclear fragment of the *Amy2B* gene alongside a fragment of a nuclear reference gene present in two diploid copy numbers (*C7orf28b*), in a multiplex polymerase chain reaction (PCR)*.* Such pre-amplification procedures have been shown to improve the sensitivity of quantitative PCR (qPCR) analysis on modern [34,35] and aDNA [36]. We followed previous recommendations to perform robust and highly accurate targeted pre-amplification in combination with qPCR [34–36].

Both fragment sequences were amplified using dog specific primers [16,18,37]:

— *Amy2B*—fragment of 76 bp: forward 5′-CCAAACCTGGACGGACATCT-3′ and reverse 5′-TATCGTTCGCATTCAAGAGCAA-3′.
— *C7orf28b-3*—fragment of 60 bp: forward 5′-GGGAAACTCCACAAGCAATCA-3′ and reverse 5′-GAGCCCATGGAGGAAATCATC-3′.

Both fragments matched in size and were designed to exclude a potential amplification bias in degraded DNA. These two pairs of primers were then mixed in a single tube. The reaction was performed in a 25 µl reaction volume containing 2.5 µl of 10× Taq buffer, 2 mM of MgCl_2_, 0.025 mg of BSA (Roche, 20 mg ml^−1^), 2.5 units of Taq polymerase (AmpliTaq Gold, Applied Biosystems), 250 µM of each dNTP (Sigma) and finally 0.5 µM of each primer. Four volumes of aDNA extract were used for each amplification: 0.5 µl, 1.0 µl, 2.0 µl and 4.0 µl. The cycling conditions were one activation step at 95°C for 2 min followed by 20 cycles of denaturation at 95°C for 30 s, then 60°C for 4 min. We systematically added two different controls in all PCR assays: an aerosol control (tube kept open throughout the manipulation to monitor airborne contaminations) and a PCR-mix control (to monitor contamination of reagents).

Amplification products were quantified using the Quantifluor® dsDNA System (Promega). This system enables the sensitive quantification of small amounts of double stranded DNA thanks to a fluorescent DNA-binding dye.

### 2.3. Quantitative polymerase chain reaction

DNA copy number variation was quantified using Multiplex TaqMan assays (primers described above). The following probes matched the target *Amy2B* gene and reference housekeeping gene *C7orf28b* [16,18,37]: *Amy2B* probe-6FAM–TTTGAGTGGCGCTGGG-MGBNFQ [33,34]; *C7orf28b-3* probe-VIC-CACCTGCTAAACAGC-MGBNFQ.

We followed the same experimental design as previously published [16,18]: the reaction was performed in a 25 µl reaction volume containing 12.5 µl of Taqman Genotyping master mix (Applied Biosystems), 0.9 µM of each primer, 0.25 µM of each probe and 2 ng of DNA. The cycling conditions were one step at 50°C for 2 min, one step at 95°C for 10 min, followed by 40 cycles of one step at 95°C for 15 s and one step at 60°C for 1 min. All reactions were run in triplicate for each sample in the same qPCR plate. We systematically added three qPCR-mix controls to monitor contamination of reagents in each assay and three aerosol controls to monitor airborne contaminations during plate preparation.

#### 2.3.1. First tests on present-day canids

In present-day wolves, the amylase copy number variation ranges from two to eight copies, with 60% of wolves bearing only two copies [17]. In order to choose a wolf reference sample to account for inter-plate variability in subsequent studies, we performed an independent qPCR on 16 wolves to evaluate the number of *Amy2B* copies (electronic supplementary material, table S2*a*), following the previous protocol. The same protocol was also used to test 16 present-day dogs (electronic supplementary material, table S2*b*).

Modern DNA work was performed in a distinct laboratory (IGDR, CNRS-UMR6290, Rennes, France). The modern DNA samples came from the biobank Cani-DNA_CRB, in IGDR-CNRS, Rennes.

#### 2.3.2. Quantitative polymerase chain reaction on ancient canids

The pre-amplification step was independently repeated before every qPCR attempt for each sample, so that each set of qPCR results (e.g. qPCR results of two different plates for the same sample) derived from independent pre-amplification. Pre-amplification controls relating to samples tested for qPCR were systematically added in the assay.

We followed the protocol described above using a present-day wolf sample as a reference to account for inter-plate variability (sample reference 8278—cani-DNA Biobank IGDR, CNRS-UMR6290, Rennes, France). Whenever possible, three positive full replicates (i.e. pre-amplification + qPCR in triplicates) were analysed for each sample and each gene.

#### 2.3.3. Quantitative polymerase chain reaction analysis

Data were analysed using the CopyCaller software (Applied Biosystems), and relative quantitative ratios (RQ) were estimated for each sample and each run. Copy numbers for each target were normalized to the reference modern wolf (sample references 8278—two amylase copies). Raw copy number data were rounded to the nearest whole number. The confidence value of the associated predicted copy number was calculated for each sample (for more details, see: https://tools.thermofisher.com/content/sfs/manuals/cms_062369.pdf).

## 3. Results

**Figure 1.**
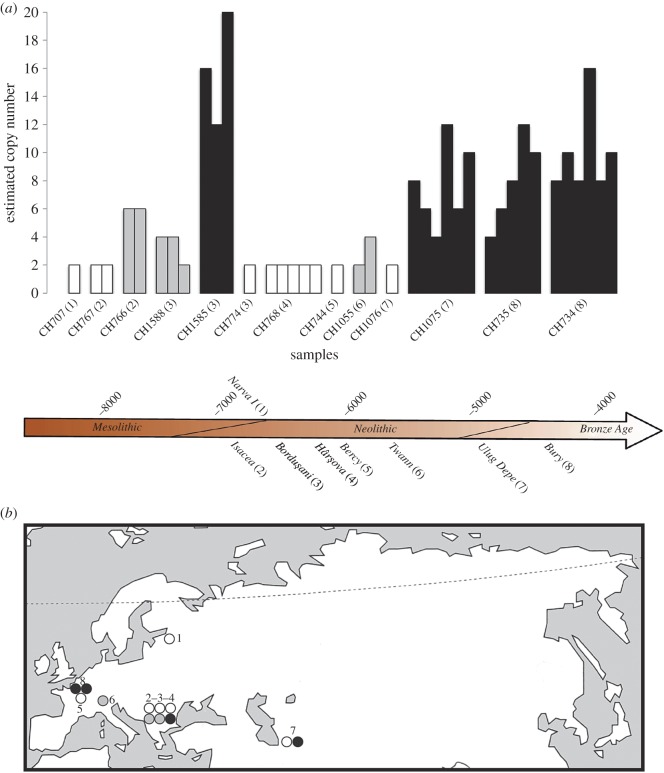
Distribution of estimated *Amy2B* diploid gene copy numbers for each specimen through space and time. (*a*) Distribution of estimated *Amy2B* diploid gene copy numbers for each specimen and replicate through time: two copies (white), two to eight copies (grey) and more than eight copies (black). (*b*) Geographical distribution of the estimated *Amy2B* diploid gene copy number variation throughout Eurasia between the Upper Palaeolithic and the Bronze Age: two copies (white), two to eight copies (grey) and more than eight copies (black).

For each aDNA sample, nuclear fragments of the *Amy2B* gene and a reference gene present in two diploid copy numbers (*C7orf28b*) were co-amplified by a qPCR procedure. In order to estimate the *Amy2B* copy number, RQ between these two genes were estimated for each sample and then normalized to the reference modern wolf (bearing two *Amy2B* copies). The protocol was tested on 16 present-day dogs and 16 present-day wolves. Results showed that the 16 wolves all evidenced two copies of the *Amy2B* gene (confidence ≥0.93; electronic supplementary material, table S2*a*), whereas the 16 dogs presented between 4 and 16 *Amy2B* copy numbers (confidence ≥ 0.93; electronic supplementary material, table S2*b*). Reproducible results (at least two full experiment replicates) were then obtained for nine ancient dogs, and a single result was obtained for four further specimens (electronic supplementary material, tables S3 and S4). RQ values varied between 0.78 and 10.04 (electronic supplementary material, table S4), indicating that the diploid *Amy2B* copy numbers varied between 2 and 20 (figure 1*a*).

Two Romanian samples (*Isaccea, Hârşova*—sites 2 and 4; figure 1*b*; electronic supplementary material, table S3) presented reproducible and concordant results indicating an estimated diploid *Amy2B* copy number of two (figure 1*a*; electronic supplementary material, table S4). Four more samples from Estonia (*Narva I*—site 1; figure 1*b*; electronic supplementary material, table S3), Romania (*Borduşani*—site 3; figure 1*b*; electronic supplementary material, table S3), Turkmenistan (*Ulug Depe*—site 7; figure 1*b*; electronic supplementary material, table S3) and north France (*Bercy*—site 5; figure 1*b*; electronic supplementary material, table S3) also bore two *Amy2B* copies, although a replicate was unobtainable (figure 1*a*; electronic supplementary material, table S4).

The three samples from Romania (*Isaccea* and *Borduşani*—sites 2 and 3; figure 1*b*; electronic supplementary material, table S3) and Switzerland (*Twann*—site 6; figure 1*b*; electronic supplementary material, table S3) carried up to six *Amy2B* copies (figure 1*a*; electronic supplementary material, table S4).

Finally, four samples from Turkmenistan (CH1075; *Ulug Depe*—site 7; figure 1*b*; electronic supplementary material, table S3), Romania (CH1585; *Borduşani*—site 3; figure 1*b*; electronic supplementary material, table S3) and north France (CH734 and CH735; *Bury*—site 8; figure 1*b*; electronic supplementary material, table S3) carried more than eight *Amy2B* copies (figure 1*a*; electronic supplementary material, table S4). Among these four samples we observed *Amy2B* copy number variations between samples and among replicates (figure 1*a*). The two samples from north France (CH735) and Turkmenistan (CH1075) evidenced between 4 and 12 estimated copy numbers. The third sample, from north France (CH734), presented between 8 and 16 *Amy2B* copy numbers. The fourth sample, from Romania (CH1585), presented the highest estimated *Amy2B* copy number, varying between 12 and 20. These four samples presented high RQ value variations between replicates (three to six replicates, with variances of 3.07, 2.11, 2.63, 4.53 for CH734, CH735, CH1075, CH1585, respectively; electronic supplementary material, table S4).

The four dogs showing *Amy2B* gene expansion (more than eight copies) came from several regions of Europe and Southwest Asia (i.e. CH1585, Borduşani, Romania, 7th millennium cal. BP; CH1075, Ulug Depe, Turkmenistan, mid- to late 5th millennium cal. BP; CH735 and Ch734, Bury, France, mid- to late 4th millennium cal. BP; see the electronic supplementary material), but no link could be established between the number of gene copy and a given geographical area. We also compared the mandibles of these four individuals (electronic supplementary material, table S1 and figure S1). The first one (CH1585) had a very short tooth row and showed oligodontia. The other three were larger but with no dental defects. No link between the number of gene copies and the morphological characteristics (i.e. size and mandible shape; electronic supplementary material, table S1 and figure S1) could be found. These results were unable to correlate the *Amy2B* gene expansion to a particular ancient dog population or morphotype.

## 4. Discussion

We obtained results for 13 of 88 samples. This success rate (15%) can be explained by aDNA degradation: (i) the estimated number of copies can differ between replicates and, therefore, must be interpreted as a minimum number of copies that could be detected and (ii) inhibition was observed in amplification curves from the majority of failed amplification attempts. We highlighted the difficulty to precisely estimate the high copy number, as it is already established that the ability to distinguish copy numbers decreases as they increase [38]. Consequently, the confidence values are often lower for high copy number samples even under optimal experimental conditions, due to the compression of the ΔCT sub distributions for high copy numbers [38].^1^ This explains some of the high copy numbers within sample variance calculated for the four individuals showing more than eight *Amy2B* copies. This phenomenon was amplified by the fact that we worked with aDNA (due to inhibition and degradation) and that two genes were targeted (reference *C7orf28b* and *Amy2B*). The pre-amplification step was necessary to restore a sufficient amount of aDNA but did not guarantee equal preservation of both targeted fragments. Enzymatic reparation of the aDNA extracts as well as droplet digital PCR (ddPCR) could be explored to improve detection efficiency. In particular, ddPCR has been shown to reduce mean coefficients of variation by 37–86% and improve reproducibility by a factor of 7 [39].

This study is, to our knowledge, the first report of qPCR being used to estimate the copy number variation from aDNA; this has led to three main issues.

### 4.1. Antiquity of the *Amy2B* gene expansion

Four of our ancient dogs displayed a high number of *Amy2B* copies (more than eight), indicating an expansion of this gene as early as the 7th millennium cal. BP in Romania (Borduşani) and the 5th millennium cal. BP in France (Bury) and Turkmenistan (Ulug Depe). These three sites correspond to a late stage in the transition to farming (Late Neolithic/Bronze Age). The *Amy2B* expansion probably allowed dogs to thrive on a starch rich diet, in comparison with the mostly carnivorous diet of wolves [18]. This constituted an important selective advantage for dogs feeding on human leftovers within a farming context. However, the scarcity of data anterior to the Neolithic does not allow us to assess whether this expansion took place before the Neolithic transition, or emerged during the Neolithic under new selection pressures related to the development of agriculture.

Currently, only a few dog lineages, such as the dingo (two copies) and the Siberian husky (three to four copies), show an unusual lack of *Amy2B* copy number. These dogs come from regions with no, or recent, agricultural practices [17,18,37]. This supports the hypothesis that the development of a dog's capability to digest starch efficiently does not result from a relaxation of the natural selection pressures related to domestication. It is more likely to result from an adaptation to the shift of human food habits during the Neolithic.

### 4.2. Persistence of a small number of copies in ancient dogs

We found ancient dogs from early farming contexts with two copies of the *Amy2B* gene at Isaccea, Hârşova, Borduşani (Romania), Ulug Depe (Turkmenistan) and Bercy (north France). Low copy number is an exceptional situation in present-day dogs and is only found in lineages associated with recent nomadic hunter–gatherers, such as the dingo and the Siberian husky. These two lineages also appear as basal on phylogenetic trees of extant dog breeds [40,41], probably as a result from a lack of recent admixture with other dog breeds due to geographical and cultural isolation [4]. Our early farming series suggests that the low *Amy2B* copy number present in their genome could stem from an ancient gene pool.

### 4.3. The *Amy2B* copy number was not fixed in early dogs

The two dog series from Borduşani and Hârşova can be considered together, as they are contemporary (mid- to late 7th millennium cal. BP) and belong to two neighbouring sites in southeast Romania. The archaeozoological series also displayed identical exploitations of animals [42,43]. The Borduşani/Hârşova set includes dogs bearing either two, two to eight or more than eight *Amy2B* copies, indicating a strong variability in the number of copies of *Amy2B* that could exist concurrently in the same population.

On a wider geographical level, our results show that dogs bearing the *Amy2B* copy number expansion came from various regions. Similarly, we found no link between the number of gene copies and specific morphotypes; though it is expected that the adaptation to a starchy diet would not only impact digestive functions but also morphological traits linked to biting and chewing (e.g. teeth, skull and mandible conformation [44]). These observations are congruent with the situation in modern dogs, where there is no fixation of the number of *Amy2B* copies in a given breed [16,17].

## 5. Conclusion

In this study, we have provided evidence for an increase of the amylase gene copy number in ancient dog genomes, with a firm a*nte quem* during the 7th millennium cal. BP in Southeastern Europe. We have demonstrated that the modern capability of numerous dogs to digest starch does not result from the selection of lineages during Classical Antiquity or the nineteenth century selection of modern breeds [19–21]; but began, at the latest, during the Neolithic, between the 10th and 7th–5th millennium cal. BP, at least in various regions of West and East Europe and Southwest Asia. We also demonstrated, on the basis of archaeological remains that the *Amy2B* copy number increase was not fixed in all dogs from Neolithic farming societies. In addition, we showed the relatively late persistence of only two copies of the *Amy2B* gene in ancient dogs, well beyond the first appearance of farming. This situation is uncommon in modern dog lineages and could not have been demonstrated without ancient data.

Further analyses, on larger samples of ancient Eurasian dogs and wolves from the Palaeolithic to the Bronze Age, would help define the precise chronology and rhythm of the *Amy2B* expansion during early dog breeding. It will also help to pinpoint the date(s) and the location(s) of the first occurrence(s) of the *Amy2B* expansion (i.e. more than eight copies).

In humans, the pattern of variation in copy numbers of the human amylase gene (*AMY1*) is consistent with a history of diet-related selection pressures [45]: higher *AMY1* copy numbers and protein levels likely improve the digestion of starchy diets. Human starch consumption increased significantly during the Neolithic transition and is correlated with a gradual increase of *AMY1* copy numbers [46,47].

The history of the *Amy2B* expansion in dogs suggests that the genes responsible for digestion in both humans and dogs probably underwent similar changes. It is reasonable to speculate that other equally compelling examples of the biocultural coevolution of human culture and dog genes, involved in metabolisms, immunity and brain processes [48,49], are yet to be identified within the dog domestication process.

## Supplementary Material

Supplementary information: archeological sites description

## Supplementary Material

Figure S1: Distribution parameters for the length of the lower tooth row (#8 in Von den Driesch, 1976 [25]; mm)

## Supplementary Material

Table S1: Measurements (in mm) on mandible for 11 ancient dog samples and dental defect record. Measurements are based on Von Den Driesh code (1976) [25].

## Supplementary Material

Table S2: Calculated and predicted Amy2B copy number and confidence score for A) 16 present-day wolves and B) 16 present-day dog samples (CaniDNA Biobank . IGDR CNRS-UMR6290)

## Supplementary Material

Table S3: Archaeological site, location, radiometric and cultural dating and aDNA results for the 13 dogs analyzed in this study.

## Supplementary Material

Table S4: Number of positive qPCR run compared to the number of independent attempts and relative Quantitative Ratio (RQ) for each independent positive run, per sample.

## Footnotes

1 Application Note: Analysis of Copy Number Variation: Design Pipeline and Validation of TaqMan® Copy Number Assays. Publication 135AP03-0.

## Data accessibility

All supporting data are made available either as electronic supplementary material or in the paper.
