# Supplementary material for: Amy2B copy number variation reveals starch diet adaptations in ancient European dogs
Source: R Soc Open Sci. 2016 Nov 9;3(11):160449. doi: 10.1098/rsos.160449 (PMC5180126; doi:10.1098/rsos.160449)
Supplement: Supplementary information: archeological sites description [file rsos160449supp1.doc]

**Archeological sites description:**

**Estonia**

**Narva :** Narva is an archaeological culture from the Neolithic period, common to Latvia, Estonia, Lithuania and Northern Belarus (7th-5th millennium cal. BP). It was described by the archaeologist N.N. Gurina during excavations in 1967 on the left bank of the River Narva. The culture is characterized by an almost complete absence of flint tools and a large number of tools made of bone, horn and wood. Sites are usually located on low hills next to rivers. The sample included in the present study is dated to the very end of the 8th millenium cal. BP.

**Romania**

**Isaccea** (departement Tulcea) is situated on the lower terrace of the Danube and dated to the Early regional Chalcolithic (Boian culture, phase Giulești, last part of the 8th millennium. cal BP) [1, 2]. Faunal remains came from refuse pits. Domestic mammals were predominant (67% of identified remains). Dogs were skinned and eaten on this site [3].

**Hârşova-tell** and **Borduşani-Popină** are neighbouring tells located approximately 20.5 km apart, on the Danube river plain in South-Eastern Romania. At both sites, Gumelniţa A2 occupations were radiocarbon dated to the second half of the 7th millennium cal BP [4, 5].

**Hârşova**-tell (Constanța county) is located on a terrace on the eastern bank of the Danube river. **Borduşani** (Ialomița county) is located on the large island of Balta Ialomiței on the Danube river. The importance of husbandry is highlighted at both sites by the clear predominance of domestic animals (>70 % of mammal remains identified to species) [6]. In Hârșova, as in Bordușani, dogs (resp. 17% and 14% of identifies remains) were skinned and eaten [7].

**Suisse**

**Twann (**Canton Berne) is a waterlogged site corresponding to a Neolithic hamlet located on the western bank of Lake Bienne [8]. It was inhabited during a period encompassing the Cortaillod and Horgen cultures (6th millennium cal. BP). The dog included in the present study comes from the Cortaillod horizon [9] and dates from the first part of the 6th millennium.

**France**

**Bercy** (Paris) is a site located on the right bank of the Seine river, downstream from the confluence with the Marne river. The site corresponds to a hamlet set on the river bank. The main occupation was dated to ca. 6,000 cal. BP and belongs to the Chasséen septentional culture (regional middle Neolithic). The canid bones included in the present study come from this cultural horizon. The faunal assemblage is dominated by domesticates. Dog bones account for less than 2% of the identified mammal bones and display no obvious trace of skinning or consumption [10].

**Bury** (Picardy Region) is a funerary monument. Most of this gallery grave dates back to the end of the Neolithic. The dogs included in the present study were from the top of the grave, from layers probably corresponding to ritual practices though not funerary activity. The dogs were radiocarbon dated to the end of the 5th millennium cal. BP, which regionally corresponds to the very beginning of the Bronze Age.

**Turkmenistan**

**Ulug Depe** is situated on the fertile belt along the northern foothills of the Kopet Dagh mountain range. This is one of the greatest proto-urban sites in Central Asia, occupied between 5,000-5,500 years BP [11]. The economy of Ulug Depe site is principally based on the exploitation of domestic and wild herbivores [11]. The samples included in the present study are dated from the mid- to late 5th millenium BP.

**Supplemental references:**

# 1. Micu C. Aşezarea neolitică de la Isaccea, punctul Suhat, jud. 2000 Tulcea, Istro-Pontica, “Muzeul tulcean la a-50-a aniversare, 1950- 2000”, Tulcea, p. 5-53.

# 2. Bălăşescu A, Radu V. 2004 Oameni şi animale. Strategii şi resurse la comunităţile preistorice Hamangia şi Boian. In Biblioteca Muzeului Naţional, Seria Cercetări Pluridisciplinare 9, Bucuresti, Editura Cetatea de Scaun, 310 p.

# 3. Bréhard S, Bălăşescu A. 2012 What’s behind the tell phenomenon? An archaeozoological approach of Eneolithic sites in Romania. Journal of Archaeological Science 39, 3167-3183.

# 4. Gillis R, et al. 2013 Sophisticated cattle dairy husbandry at Borduşani-Popina (Romania, fifth millennium BC): the evidence from complementary analysis of mortality profiles and stable isotopes. World Archaeology 45, 447-472.

# 5. Bălăşescu A, Moise D, Radu V. 2005 The palaeoeconomy of Gumelnița communities on the territory of Romania. Cultură și civilizație la Dunărea de Jos 22, 167–200.

# 6. Lazăr C, Mărgărit M, Bălășescu A. 2016 Dogs, jaws, and other stories: two symbolic objects made of dog mandibles from Southeastern Europe, Journal of Field Archaeology 41, 1, p. 101- 117

# 7. Furger AR, & Hartmann F. 1983 Vor 5000 Jahren, so lebten unsere Vorfahren in der Jungsteinzeit. Bern, Paul Haupt Verlag.

# 8. Becker C & Johansson F. 1981 Die Neolithischen Ufersiedlungen von Twann. Staatlicher Lehrmittleverlag Bern.

# 9. Tresset A. 1993 Quelques aspects de l’approvisionnement carné en contexte Chasséen septentrional à Bercy. Rapport d’analyse. Service Régional de l’Archéologie d’Ile de France.

# 10. Lecomte O. 2007 An Iron Age Urban Settlement Revealed by Magnetic Survey: The Case of Ulug depe [Turkmenistan] », in M. Posselt, B. Zickgraft, C. Dobiat (eds.), Geophysics and excavation. Deployment and utilization of non-destructive prospection methods in Archaeology, p. 99-111.

# 11. Mashkour M. 2013 Sociétés pastorales et économies de subsistance au nord-est de l’Iran et au sud du Turkménistan. Cahiers d’Asie centrale
