# Supplementary material for: Amy2B copy number variation reveals starch diet adaptations in ancient European dogs
Source: R Soc Open Sci. 2016 Nov 9;3(11):160449. doi: 10.1098/rsos.160449 (PMC5180126; doi:10.1098/rsos.160449)
Supplement: Figure S1: Distribution parameters for the length of the lower tooth row (#8 in Von den Driesch, 1976 [25]; mm) [file rsos160449supp2.doc]

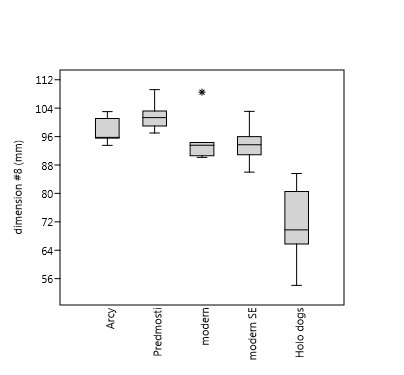


CH1075

CH734

CH735

CH1585

**Figure S1:** Distribution parameters for the length of the lower tooth row (#8 in Von den Driesch, 1976 [25]; mm)

*Arcy: Pleistocene wolves from Arcy-sur Cure (n=5, [23]), Předmostí: Pleistocene wolves from Předmostí (n=28, [26]), modern: modern Eurasian wolves from the Muséum National d’Histoire Naturelle (n=8, [23]), modern SE: modern wolves from South-Eastern Europe (n=39, [27]), Holo dogs: Holocene dogs considered in this study (n=10).*

*The four samples discussed more precisely in the results section are represented by*
