# Supplementary material for: Amy2B copy number variation reveals starch diet adaptations in ancient European dogs
Source: R Soc Open Sci. 2016 Nov 9;3(11):160449. doi: 10.1098/rsos.160449 (PMC5180126; doi:10.1098/rsos.160449)
Supplement: Table S1: Measurements (in mm) on mandible for 11 ancient dog samples and dental defect record. Measurements are based on Von Den Driesh code (1976) [25]. [file rsos160449supp3.docx]

**Table S1**: Measurements (in mm) on mandible for 11 ancient dog samples and dental defect record. Measurements are based on Von Den Driesh code (1976) [25]. Blank: data not available. Only measurements for individuals providing aDNA results are reported.

|  | **Sample references** | | | | | | | | | | |
| --- | --- | --- | --- | --- | --- | --- | --- | --- | --- | --- | --- |
| **Point references on mandible** | CH767 | CH766 | CH1588 | CH1585 | CH768 | CH744 | CH1055 | CH1076 | CH1075 | CH735 | CH734 |
| Site | Isaccea | Isaccea | Bordușani | Bordușani | Hârșova | Bercy | Twann | Ulug Depe | Ulug Depe | Bury | Bury |
| 1 | 113.94 |  | 131 | 105.5 | 113.97 | 120 |  |  |  |  | 156.65 |
| 2 | 112.36 |  | 130.5 | 108.3 | 112.58 | 120.8 |  |  |  |  | 156.3 |
| 3 | 108.54 |  | 125 | 101 | 107.55 | 115.5 |  |  |  |  | 147.83 |
| 4 | 101.25 |  | 112.58 | 91 | 102.69 | 105 |  |  |  | 140.3 | 134.47 |
| 5 | 97.01 |  | 107.46 | 86.9 | 97.36 | 101 | 95.1 |  |  | 133.99 | 126.23 |
| 6 | 100.86 |  | 111.13 | 94.3 | 102.03 | 106.5 |  |  |  | 142.63 | 133.18 |
| 7 | 67.24 |  | 74.41 | 58.1 | 67.93 | 71.8 | 69.74 | 87.93 | 88.1 | 88.12 | 83.22 |
| 8 | 62.49 |  | 69.72 | 54.1 | 65.77 | 68.6 | 66.35 | 83.45 | 85.6 | 80.53 | 76.75 |
| 9 | 54.62 |  | 64.69 | Oligodontia P2 | 60.95 | P2 fallen | 62.57 | 78.04 | 79.5 | 74.61 | 72.08 |
| 10 | 29.8 |  | 32.03 | 25.7 | 31.61 | 35.2 | 33.06 | 44.11 | 40.5 | 36.43 | 34.85 |
| 11 | 32.4 | 37.02 | 36.81 | 28.8 | 34.19 | 34.2 | 29.62 | 38.7 | 43.8 | 45.99 | 40.4 |
| 12 | 28.97 | 31.48 | 32.04 | Oligodontia P2 | 29.31 | P2 fallen | 35.16 | 41.24 | 38.8 | 39.3 | 35.67 |
| 13L |  | 22.16 | 20.15 | Absent | 19.88 | Absent | 21.43 | 25.11 | Absent | 22.37 | 20.58 |
| 13B |  | 8.58 | 8.28 | Absent | 8.56 | Absent | 8.38 | 10.54 | Absent | 9.69 | 9.09 |
| 14 | 17.58 | 21.88 | 20.09 | 17.8 | 19.26 | 21.9 | 20.15 | 23.48 | 25.1 | 20.4 | 18.48 |
| 15L |  |  | 8.54 | Absent | 8.46 | Absent | 9.19 | 10.41 | Absent | 9.72 | 9.81 |
| 15B |  |  | 5.8 | Absent | 5.97 | Absent | 7.12 | 8.11 | Absent | 7.32 | 7.32 |
| 16L |  |  |  | Absent |  | Absent |  |  | Absent |  |  |
| 16B |  |  |  | Absent |  | Absent |  |  | Absent |  |  |
| 17 | 9.69 | 10.4 | 10.23 | 9.8 | 10.31 | 11.5 | 10.34 | 14.74 | 13.5 | 12.81 | 12.12 |
| 18 | 47.65 |  | 51.14 | 44.6 | 43.58 |  |  |  |  |  | 63.45 |
| 19 | 21.36 | 22.93 | 20.53 | 19.8 | 20.1 | 22.3 | 19.31 | 30.78 | 32 | 28.11 | 27.08 |
| 20 | 17.32 | 18.28 | 18.17 | 15.4 | 17.6 | 16.7 | 17.08 | 24.54 | 26.5 | 22.47 | 20.42 |
| Oligodontia |  |  | No | P2, M3 |  | P2 fallen, filled alveole |  |  | No | No | No |
