# Supplementary material for: Amy2B copy number variation reveals starch diet adaptations in ancient European dogs
Source: R Soc Open Sci. 2016 Nov 9;3(11):160449. doi: 10.1098/rsos.160449 (PMC5180126; doi:10.1098/rsos.160449)
Supplement: Table S2: Calculated and predicted Amy2B copy number and confidence score for A) 16 present-day wolves and B) 16 present-day dog samples (CaniDNA Biobank . IGDR CNRS-UMR6290) [file rsos160449supp4.docx]

**Table S2:** Calculated and predicted *Amy2B* copy number and confidence score for A) 16 present-day wolves and B) 16 present-day dog samples (CaniDNA Biobank . IGDR CNRS-UMR6290)

Confidence : The confidence value of the associated predicted copy number (https://tools.thermofisher.com/content/sfs/manuals/cms_062369.pdf)

**A**

| **Sample Reference**  **(CaniDNA BioBank)** | **Copy Number Calculated** | **Copy Number Predicted** | **Confidence** |
| --- | --- | --- | --- |
| 6813 | 1.02 | 2 | > 0.99 |
| 6916 | 0.90 | 2 | > 0.99 |
| 6917 | 1.31 | 2 | 0.93 |
| 7030 | 1.08 | 2 | > 0.99 |
| 7031 | 0.95 | 2 | > 0.99 |
| 7032 | 0.97 | 2 | > 0.99 |
| 7033 | 1.12 | 2 | > 0.99 |
| 7035 | 0.96 | 2 | > 0.99 |
| 7036 | 1.18 | 2 | 0.99 |
| 7037 | 0.89 | 2 | > 0.99 |
| 7056 | 1.09 | 2 | > 0.99 |
| 7057 | 0.99 | 2 | > 0.99 |
| 7058 | 0.95 | 2 | > 0.99 |
| 7180 | 0.85 | 2 | > 0.99 |
| 7269 | 1.17 | 2 | 0.99 |
| 8278 | 0.84 | 2 | > 0.99 |
|  |  |  |  |

**B**

| **Sample Reference**  **(CaniDNA BioBank)** | **Copy Number Calculated** | **Copy Number Predicted** | **Confidence** |
| --- | --- | --- | --- |
| 8278 - wolf | 1.00 | 2 | > 0.99 |
| 7513 - Malamute | 3.88 | 8 | 0.94 |
| Bandit - Malamute | 5.88 | 12 | > 0.99 |
| Jenny - Malamute | 4.11 | 8 | 0.99 |
| Akita americain | 1.97 | 4 | 0.97 |
| Alaska- Samoyede | 4.87 | 10 | > 0.99 |
| Canelle - Beagle | 4.25 | 8 | > 0.99 |
| DAIKA - Boxer | 4.59 | 9 | > 0.99 |
| Djazy - Eurasier | 3.85 | 8 | 0.93 |
| Vivaldi - Eurasier | 4.00 | 8 | 0.97 |
| Donald - Chihuahua | 5.37 | 11 | > 0.99 |
| Ectasy- Akita | 1.83 | 4 | 0.99 |
| Farell - Husky | 1.95 | 4 | 0.97 |
| Fute - Beagle | 4.16 | 8 | 0.99 |
| Holifey - Saluki | 4.30 | 9 | > 0.99 |
| Shamsul - Saluki | 7.29 | 15 | > 0.99 |
| Shererazade - Saluki | 8.01 | 16 | > 0.99 |
