# Supplementary material for: Amy2B copy number variation reveals starch diet adaptations in ancient European dogs
Source: R Soc Open Sci. 2016 Nov 9;3(11):160449. doi: 10.1098/rsos.160449 (PMC5180126; doi:10.1098/rsos.160449)
Supplement: Table S3: Archaeological site, location, radiometric and cultural dating and aDNA results for the 13 dogs analyzed in this study. [file rsos160449supp5.doc]

**Table S3**: Archaeological site, location, radiometric and cultural dating and aDNA results for the 13 dogs analyzed in this study.

| **Archeological Site / Country** | **Date obtained directly on dog bones (cal. BP)** | **Date derived from other remains (cal. BP)** | **Chronological periods derived from cultural attributions** | **Cultural period** | **Results obtained /  Individuals tested for the considered archeological site** |
| --- | --- | --- | --- | --- | --- |
| *Bury / France* |  |  | 4400-4200 | **EARLY BRONZE AGE**  **France** | **2/2** |
|  |  |  |  |  |  |
| *Ulug Depe / Turkmenistan* |  | [5450-3950] |  | **BRONZE AGE Turkmenistan** | **2/3** |
|  |  |  |  |  |  |
| *Twann / Switzeland* |  |  | 6150-5400 | **NEOLITHIC**  **Chasséen Culture** | **1/5** |
| *Bercy / France* |  | [6311-5875] |  |  | **1/3** |
|  |  |  |  |  |  |
| *Hârșova / Romania* |  |  | 6500-5900 | **CHALCOLITHIC Boian and Gumelnița culture Romania** | **1/4** |
| *Bordușani / Romania* | [6798-6674] |  | 6798-5900 |  | **3/17** |
| *Isaccea / Romania* |  |  | 7000-6600 |  | **2/7** |
|  |  |  |  |  |  |
| *Narva I / Estonia* | [7161-5583] |  |  | **MESOLITHIC**  **Narva Culture Estonia** | **1/4** |
|  |  |  |  |  |  |
