# Supplementary material for: Amy2B copy number variation reveals starch diet adaptations in ancient European dogs
Source: R Soc Open Sci. 2016 Nov 9;3(11):160449. doi: 10.1098/rsos.160449 (PMC5180126; doi:10.1098/rsos.160449)
Supplement: Table S4: Number of positive qPCR run compared to the number of independent attempts and relative Quantitative Ratio (RQ) for each independent positive run, per sample. [file rsos160449supp6.docx]

**Table S4:** Number of positive qPCR run compared to the number of independent attempts and relative Quantitative Ratio (RQ) for each independent positive run. per sample.

A to P: independent qPCR runs.

*: For these samples. a single attempt was positive despite a great number of tests.

| **Sample  Reference** | **Country** | **Site** | **Number of  positive amplifications / Number of attempts** | **Variance** | **A** | **B** | **C** | **D** | **E** | **F** | **G** | **H** | **I** | **J** | **K** | **L** | **M** | **N** | **O** | **P** |
| --- | --- | --- | --- | --- | --- | --- | --- | --- | --- | --- | --- | --- | --- | --- | --- | --- | --- | --- | --- | --- |
| CH734 | France | Bury | 6/14 | 3.07 |  | 4.48 |  | 4.59 |  | 3.6 |  |  | 8.4 |  |  |  |  |  |  | 3.90 5.49 |
| CH735 | France | Bury | 5/18 | 2.11 | 2.46 |  |  |  | 3.21 |  |  |  | 3.79 |  | 6.26 | 4.57 |  |  |  |  |
| CH1075 | Turkmenistan | Ulug depe | 6/12 | 2.63 | 3.66   2.61 | 2.02 | 6.42 |  | 3.19 |  |  |  |  |  |  |  |  |  |  | 4.95 |
| CH1076 | Turkmenistan | Ulug depe | 1/16* |  |  |  | 0.78 |  |  |  |  |  |  |  |  |  |  |  |  |  |
| CH1055 | Switzerland | Twann | 2/17 | 0.52 |  |  |  |  |  |  |  |  |  | 1.03 |  |  |  | 2.05 |  |  |
| CH744 | France | Bercy | 1/10* |  |  |  |  |  |  |  |  | 1.37 |  |  |  |  |  |  |  |  |
| CH768 | Romania | Hârșova | 5/7 | 0.05 |  | 1.01 | 1.37 | 1.29 0.83 |  | 1.25 |  |  |  |  |  |  |  |  |  |  |
| CH774 | Romania | Bordușani | 1/26* |  |  |  |  |  |  |  | 1.02 |  |  |  |  |  |  |  |  |  |
| CH1585 | Romania | Bordușani | 3/14 | 4.53 |  |  |  |  |  |  |  |  | 7.57 |  | 5.8 | 10.04 |  |  |  |  |
| CH1588 | Romania | Bordușani | 3/16 | 0.74 |  |  |  |  |  |  |  |  |  |  |  |  | 2.34 | 1.92 | 0.68 |  |
| CH766 | Romania | Isaccea | 2/16 | 0.08 |  |  |  |  |  |  |  |  | 2.92 |  |  |  |  | 2.51 |  |  |
| CH767 | Romania | Isaccea | 2/13 | 0.002 |  | 0.91 | 0.98 |  |  |  |  |  |  |  |  |  |  |  |  |  |
| CH707 | Estonia | Narva I | 1/18* |  |  |  |  |  |  |  | 1.43 |  |  |  |  |  |  |  |  |  |
